# Immunogenicity of an Oil-in-Water Emulsion Containing *Hafnia Alvei*-Derived Lipopolysaccharide, with TLR4 and Dectin-2 Agonist Activity In Vitro

**DOI:** 10.3390/vaccines14070557

**Published:** 2026-06-25

**Authors:** Ri Ra Hong, Eun Ji Lee, Ji Hee Kwon, Sun Woo Im, Yeji Nam, Hyun-Tae Son, Eunhye Yoo, Hyung Tae Lee

**Affiliations:** 1 Eutilex Co., Ltd., Gasan Digital 1-Ro 58, Geumcheon-gu, Seoul 08591, Republic of Korea; 2 College of Veterinary Medicine, Kangwon National University, Chuncheon 24341, Republic of Korea; 3 KELGEN Inc., Hana-ro 820-120, Iksan-si 54531, Republic of Korea

**Keywords:** *Hafnia alvei*, lipopolysaccharide (LPS), TLR4, Dectin-2, vaccine adjuvant, oil-in-water (O/W) emulsion

## Abstract

Background: Lipopolysaccharide (LPS) functions as a Toll-like receptor 4 (TLR4) agonist that triggers innate immunity; however, structural variations between pathogenic and commensal bacteria distinctly influence its immunostimulatory profile. This study evaluated the immunostimulatory activity of LPS derived from the commensal bacterium *Hafnia alvei* and explored its potential as an exploratory vaccine adjuvant. Methods: Cytokine induction was evaluated in immune cells across diverse host species, and receptor activation was assessed via reporter assays. To investigate in vivo immunogenicity and preliminary tolerability, *H. alvei* LPS was formulated into a prototype oil-in-water (O/W) emulsion utilizing ovalbumin (OVA) as a model antigen. Results: LPS from *H. alvei* strain BA2000346 exhibited immunostimulatory activity comparable to that of *Escherichia coli*, while inducing greater TNF-α expression than pathogenic *Salmonella* and *Pseudomonas* strains. Distinct from *E. coli* LPS, it demonstrated the capacity to activate both TLR4 and the mannose-recognizing Dectin-2 receptor in reporter systems. This cytokine induction was consistent across various strains and host species. Furthermore, the prototype O/W emulsion formulation enhanced antigen-specific humoral and cellular immune responses while demonstrating preliminary tolerability based on body-weight monitoring and visual clinical observation. Conclusions: *H. alvei*-derived LPS exhibits TLR4 and Dectin-2 agonist activity in vitro. When synergized with an O/W emulsion delivery system, it provides a preliminary indication of cross-species stimulatory potential and supports further investigation as a hypothesis-generating platform for future vaccine adjuvant development.

## 1. Introduction

Adjuvants are essential components of modern vaccines, critical for enhancing the magnitude and duration of the immune response to antigens such as inactivated viruses, recombinant proteins, or synthetic peptides, which typically exhibit limited intrinsic immunogenicity. Aluminum salts (Alum), among the earliest and most extensively utilized adjuvants, effectively promote humoral immunity by driving Type 2 helper T (Th2) responses. However, they demonstrate limited efficacy in stimulating Type 1 helper T (Th1) responses and cytotoxic T cell activity, which are imperative for protection against intracellular viral and bacterial pathogens [1,2,3,4]. Historically, early-generation adjuvants—including Alum and conventional oil-in-water (O/W) emulsions such as MF59 and AS03—functioned predominantly as delivery vehicles to improve antigen uptake rather than as direct immunomodulators [5,6].

To address these limitations, contemporary adjuvant research has shifted toward developing next-generation immunostimulatory agents that directly activate pattern recognition receptors (PRRs) on innate immune cells. This includes agonists for Toll-like receptors (TLRs), C-type lectin receptors (CLRs), and the cGAS-STING pathway [6,7]. Among these, TLR agonists have been the most extensively investigated. Nevertheless, the clinical translation of these candidates remains challenging; to date, only a few TLR-based adjuvants have been licensed for human use, notably Monophosphoryl Lipid A (MPL, a TLR4 agonist by GSK) and CpG ODN1018 (a TLR9 agonist by Dynavax) [6,7,8].

Lipopolysaccharide (LPS), a major structural component of the Gram-negative bacterial outer membrane, is a well-recognized TLR4 agonist with well-documented adjuvant properties. Due to its inherent endotoxicity, LPS derived from pathogenic bacteria is frequently chemically modified into detoxified lipid A (e.g., MPL) to ensure tolerability for clinical application [9]. Importantly, structural variations in the O-antigen and lipid A moieties across different bacterial species significantly dictate their immunostimulatory profiles and binding affinities [10,11,12]. Emerging evidence suggests that LPS from intestinal commensal bacteria, unlike that from highly pathogenic strains, can stimulate differentiated immune responses with inherently lower toxicity and distinct pathway selectivities. This highlights their unexploited potential as novel, naturally attenuated adjuvant candidates [11].

*Hafnia alvei* is a versatile Gram-negative bacterium widely distributed across diverse habitats. While primarily recognized as a commensal organism in the digestive tracts of humans and various animals, it is also ubiquitous in the general environment, including soil, water, and various food products. This prevalent commensal and environmental nature implies a long-standing evolutionary interaction with the host immune system, potentially offering a distinct immunomodulatory profile compared to highly virulent pathogens. Building on this rationale, we hypothesized that LPS derived from the commensal bacterium *H. alvei* could serve as an exploratory immunostimulatory component for vaccine formulations. Furthermore, to maximize clinical translatability and ensure structural stability of the co-administered antigens, we hypothesized that formulating this LPS within a prototype oil-in-water (O/W) emulsion would provide a delivery system that facilitates antigen uptake and immune activation.

To test these hypotheses, we systematically evaluated cytokine induction by LPS from multiple *H. alvei* strains across human, murine, and porcine immune cells. Subsequently, *H. alvei* LPS was formulated into a prototype O/W emulsion, and its exploratory adjuvant potential—encompassing both humoral and cellular immunity—was evaluated in a proof-of-concept in vivo murine model.

## 2. Materials and Methods

### 2.1. Cell Culture

Mouse splenocytes were isolated from C57BL/6 mice and cultured in RPMI 1640 medium (Welgene, Gyeongsan, Republic of Korea) supplemented with 10% fetal bovine serum (FBS; HyClone, Logan, UT, USA) and 1% penicillin/streptomycin (P/S; Gibco, Grand Island, NY, USA) at 37°C in a humidified 5% CO_2_ atmosphere.

The human monocytic THP-1 cell line was obtained from the American Type Culture Collection (ATCC, Manassas, VA, USA) and maintained in RPMI 1640 medium supplemented with 10% FBS, 1% P/S, and 0.05 mM 2-mercaptoethanol (Gibco) at 37°C in a humidified 5% CO_2_ incubator. To induce differentiation into macrophages, THP-1 monocytes were treated with 100 ng/mL phorbol 12-myristate 13-acetate (PMA; Sigma-Aldrich, St. Louis, MO, USA) for 48 h.

Porcine peripheral blood mononuclear cells (PBMCs) and alveolar macrophages (PAMs) were provided by the Animal and Plant Quarantine Agency (Gimcheon, Republic of Korea) and cultured in RPMI 1640 medium containing 10% FBS and 1% P/S at 37 °C in a humidified 5% CO_2_ atmosphere.

### 2.2. Bacterial Strains

*H. alvei* strains BA2000103 and BA2000346 were obtained from the Korea Veterinary Culture Collection (Gimcheon, Republic of Korea). *H. alvei* strains BAA-2768, ATCC 25927, and ATCC 51815 were purchased from ATCC. *Escherichia coli* K12, *Salmonella enterica* subsp. *enterica* serovar Typhimurium LT2, and *Pseudomonas aeruginosa* PAO1 were provided by Prof. Jang Won Yoon (Kangwon National University, Chuncheon, Republic of Korea).

### 2.3. Lipopolysaccharide Extraction

Bacterial strains were cultured in Tryptic Soy Broth (TSB; BD Difco, Sparks, MD, USA) at 37 °C with shaking at 230 rpm for 20 h. LPS was extracted from cultures containing approximately 1 × 10^9^–1 × 10^10^ colony-forming units (CFUs) using an LPS extraction kit (Intron Biotechnology, Seongnam, Republic of Korea) according to the manufacturer’s instructions. The extraction kit is based on a modified phenol/chloroform extraction method. During the extraction process, cellular proteins and nucleic acids are effectively denatured and partitioned into the organic phase or precipitate at the interphase, while lipopolysaccharides remain highly soluble in the aqueous phase. Because of this efficient phase-separation mechanism, an additional Proteinase K digestion step was not strictly required in this protocol [13,14]. LPSs from *E. coli* O111:B4 (Cat. No.: tlrl-eblps), *Porphyromonas gingivalis* (Cat. No.: tlrl-pglps) and *Salmonella minnesota* R595 (Cat. No.: tlrl-smlps) were purchased from InvivoGen (Hong Kong, China). The absolute concentration of the purified LPS was determined by measuring the dry weight of the pellet. The purity of the extracted LPS was routinely verified using a Pierce Silver Stain Kit (Thermo Fisher Scientific Inc., Waltham, MA, USA), as shown in Supplementary Figure S1.

### 2.4. In Vitro Cytokine Assay

Mouse splenocytes (1 × 10^6^ cells/mL), THP-1-derived macrophages (1 × 10^6^ cells/mL), porcine PBMCs (4 × 10^5^ cells/mL) and PAMs (1 × 10^6^ cells/mL) were stimulated with LPS (100 ng/mL) at 37 °C in a 5% CO_2_ atmosphere for 24 h. Supernatants were collected, and cytokine levels (IL-2, IL-6, IL-10, TNF-α, and IFN-γ) were measured using a Cytometric Bead Array (CBA; BD Biosciences, San Jose, CA, USA) or an ELISA kit (Thermo Fisher Scientific Inc.) according to the manufacturer’s instructions.

### 2.5. TLR4 and Dectin-2 Reporter Assay

HEK-Blue^TM^ human TLR4 (hTLR4) and mouse Dectin-2 (mDectin-2) reporter cells (InvivoGen) were cultured in Dulbecco’s Modified Eagle’s Medium (DMEM, high glucose) supplemented with 10% FBS and 1% P/S. Normocin (0.1 mg/mL) was added for hTLR4 cells, and puromycin (1 μg/mL) for mDectin-2 cells. Cells were stimulated with LPS from *E. coli* O111:B4 and *H. alvei* strain BA2000346 for 24 h. Reporter activity was quantified by measuring absorbance at 655 nm.

### 2.6. Adjuvant Formulation and In Vivo Immunization

LPS (20 μg/mL) from *H. alvei* strains was formulated into a prototype squalene-based O/W emulsion. The prototype O/W emulsion is based on a proprietary mixture comprising squalene as the core oil phase, stabilized by key excipients including glycerol and polysorbate 80. The O/W emulsion was prepared by emulsifying the oil phase with the aqueous phase utilizing microfluidization. Due to proprietary restrictions regarding the complete formulation, further specific manufacturing parameters cannot be fully disclosed. The basic physicochemical characterization of the formulated emulsion demonstrated a mean pH of 6.5 ± 0.5 and an average particle size of 101.39 ± 2.14 nm. Ovalbumin (OVA; 10 μg/dose, EndoFit^TM^, InvivoGen) was mixed with the adjuvant at a 1:1 (*v*/*v*) ratio to a final injection volume of 100 μL.

Six-week-old female C57BL/6N mice (Orient Bio Inc., Seongnam, Republic of Korea) were acclimated for 7 days. During this period and throughout the entire experimental duration, the mice were housed under specific pathogen-free (SPF) conditions, maintained on a 12 h light/dark cycle, and provided ad libitum access to standard chow and water. Prior to immunization, the animals were randomly assigned to experimental groups based on their baseline body weights. The mice were immunized intramuscularly twice at two-week intervals. To ensure data integrity and minimize observer bias, a strict separation of duties and blinding protocol was implemented. The investigators responsible for in vivo animal handling, immunizations, and clinical monitoring were blinded to the specific compositions of the test formulations, which were provided strictly as coded groups. Following the immunizations, the mice were monitored daily through visual inspection for any signs of local injection-site reactogenicity and systemic distress, alongside routine body-weight measurement. Serum samples were collected on days 0, 13, and 28. Splenocytes were harvested at 14 days after the second immunization for T-cell response analysis. Furthermore, all downstream immunological evaluations, including tissue processing, ELISA/ELISpot quantification, and flow cytometry data acquisition, along with statistical analysis, final data interpretation, and figure preparation, were performed independently by researchers who were entirely blinded to the in vivo treatment allocations.

### 2.7. Antibody Analysis

OVA-specific total IgG, IgG1, and IgG2c levels were measured by indirect ELISA. Briefly, 96-well Maxisorp plates (Nunc, Rochester, NY, USA) were coated with OVA (1 μg/well) overnight at 4 °C, blocked with 1% (*w*/*v*) BSA in PBS, and incubated with diluted serum samples for 2 h at 37 °C. Bound antibodies were detected using HRP-conjugated secondary antibodies. The reaction was developed with TMB substrate and stopped with 2 M H_2_SO_4_, and absorbance was measured at 450 nm using a Multiskan GO UV/Vis spectrophotometer (Thermo Fisher Scientific Inc.).

### 2.8. Intracellular Cytokine Staining (ICS) Assay

Splenocytes (2 × 10^6^ cells/well) were stimulated with OVA (50 μg/mL) for 15 h, with Brefeldin A added at a 1:1000 dilution during the last 10 h. Following stimulation, cells were stained for surface markers (CD4 and CD8) and intracellular cytokines (IFN-γ, IL-2, and TNF-α). Data acquisition was performed using a BD FACS Celesta flow cytometer, and analysis was conducted with FlowJo software v10.0 (BD Biosciences). Single-color compensation controls were used to calculate the compensation matrix. To accurately establish the positive gating boundaries for cytokine-producing CD4^+^ and CD8^+^ T-cell populations, isotype-matched control antibodies were utilized as negative controls, and cells stimulated with Concanavalin A (ConA) were employed as biological positive controls.

### 2.9. Enzyme-Linked Immunospot (ELISpot) Assay

OVA-specific IFN-γ production was measured using a Mouse IFN-γ ELISpot assay kit (Mabtech, Stockholm, Sweden) following the manufacturer’s protocol. Splenocytes (2.5 × 10^5^ cells/well) were stimulated with OVA_257–264_ (SIINFEKL, 10 μg/mL) (Peptron, Daejeon, Republic of Korea) for 20 h. Spot-forming units (SFUs) were quantified using a BioSpot S5 analyzer (Cellular Technology Ltd., Shaker Heights, OH, USA).

### 2.10. Statistical Analysis

Due to the relatively small sample sizes inherent to the experimental design (e.g., *n* = 4 per group for in vivo studies, and *n* = 2 or 3 for specific in vitro assays), non-parametric statistical methods were strictly applied to ensure robust and accurate data interpretation. Data were analyzed using GraphPad Prism version 7 (GraphPad Software, Inc., San Diego, CA, USA) and are presented as mean ± standard deviations (SDs). Although median and interquartile ranges are often considered for small non-parametric datasets, mean ± SD was retained in this study to maintain descriptive consistency with established conventions in the immunological adjuvant literature and to transparently illustrate the spread of individual biological variations within the small sample size.

Multiple-group comparisons were evaluated using the Kruskal–Wallis test, followed by the Mann–Whitney test for post hoc pairwise comparisons. Due to the exploratory, proof-of-concept nature of this study, these post hoc pairwise comparisons were conducted without corrections for multiple comparisons. Exact *p*-values are provided where possible. A *p*-value of <0.05 was considered statistically significant.

## 3. Results

### 3.1. LPS from Commensal Bacterium H. alvei Elicits Substantial TNF-α Production in Murine Splenocytes

**Figure 1:**
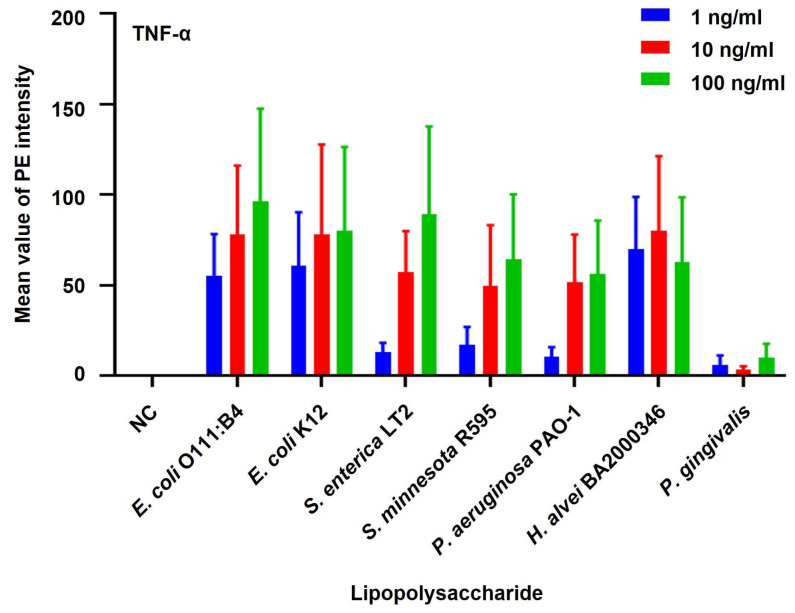
Immune cell-activating effects of lipopolysaccharides derived from various Gram-negative bacteria. Mouse splenocytes (1 × 10^6^ cells/mL) were stimulated with LPS (1–100 ng/mL) for 24 h. TNF-α levels in the culture supernatants were measured using a CBA assay. Data are presented as mean ± SD (*n* = 3).

To investigate whether LPS derived from the commensal bacterium *H. alvei* can effectively activate innate immune cells, murine splenocytes were stimulated for 24 h with LPS (1–100 ng/mL) isolated from various Gram-negative bacteria, including *H. alvei* strain BA2000346. TNF-α production was quantified as a primary indicator of immune activation. As shown in Figure 1, LPS from *Porphyromonas gingivalis* induced the lowest TNF-α expression among all tested strains. In contrast, LPS from *H. alvei* strain BA2000346 elicited substantial TNF-α production, reaching levels comparable to those induced by LPS from highly pathogenic bacteria such as *Salmonella minnesota* R595, *S. typhimurium* LT2, *Pseudomonas aeruginosa* PAO1, and *Escherichia coli* O111:B4. These results indicate that *H. alvei* LPS functions as an exploratory immunostimulant, exerting immune-activating effects that rival those of pathogenic Gram-negative strains but within a commensal context.

### 3.2. H. alvei Strain BA2000346 LPS Exhibits TLR4 and Dectin-2 Agonist Activity In Vitro

While LPS is canonically recognized as a TLR4 agonist, specific structural variations in its O-antigen composition can facilitate recognition by diverse pattern recognition receptors (PRRs) [6]. Notably, the LPS O-antigens of several Gram-negative bacteria, including *H. alvei*, contain α-linked mannose—a well-documented ligand for the C-type lectin receptor, Dectin-2 [15].

**Figure 2:**
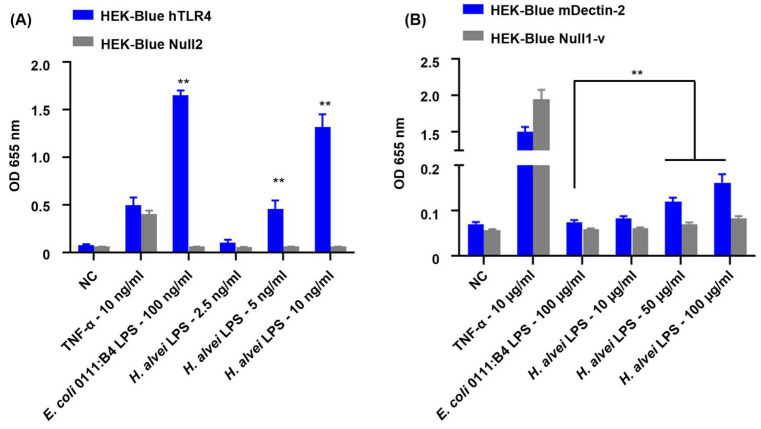
LPS from *H. alvei* strain BA2000346 activates both TLR4 and Dectin-2. (**A**) HEK-Blue^TM^ human TLR (hTLR4) and (**B**) mouse Dectin-2 (mDectin-2) reporter cells were stimulated with LPS from *E. coli* O111:B4 or *H. alvei* BA2000346 for 24 h. Reporter activity was measured by absorbance at 655 nm. Data are shown as mean ± SD (*n* = 6). Statistical significance was assessed using the Mann–Whitney test; ** *p* < 0.01. Comparisons were made against the negative control for hTLR4 and against *E. coli* O111:B4 for mDectin-2.

We first assessed the capacity of *H. alvei* strain BA2000346 LPS to activate TLR4. Despite utilizing a 10-fold lower concentration (10 ng/mL) compared to *E. coli* O111:B4 LPS (100 ng/mL), BA2000346 LPS induced comparable TLR4 activation with no statistically significant difference (Figure 2A). Subsequently, we evaluated Dectin-2 activation. Significantly, BA2000346 LPS induced approximately 2.14-fold greater Dectin-2 activation than *E. coli* O111:B4 LPS (Figure 2B, *p* < 0.01). These findings demonstrate the intrinsic capacity of BA2000346 LPS to exhibit both TLR4 and Dectin-2 agonist activity in reporter systems.

### 3.3. H. alvei LPS Induces Substantial Cytokine Responses Across Human, Murine, and Porcine Immune Cells

Given that structural variations in LPS (e.g., O-antigen, core oligosaccharide, and lipid A) can occur even among strains of the same species and significantly alter immunostimulatory profiles [10], we systematically compared cytokine induction across multiple *H. alvei* strains.

**Figure 3:**
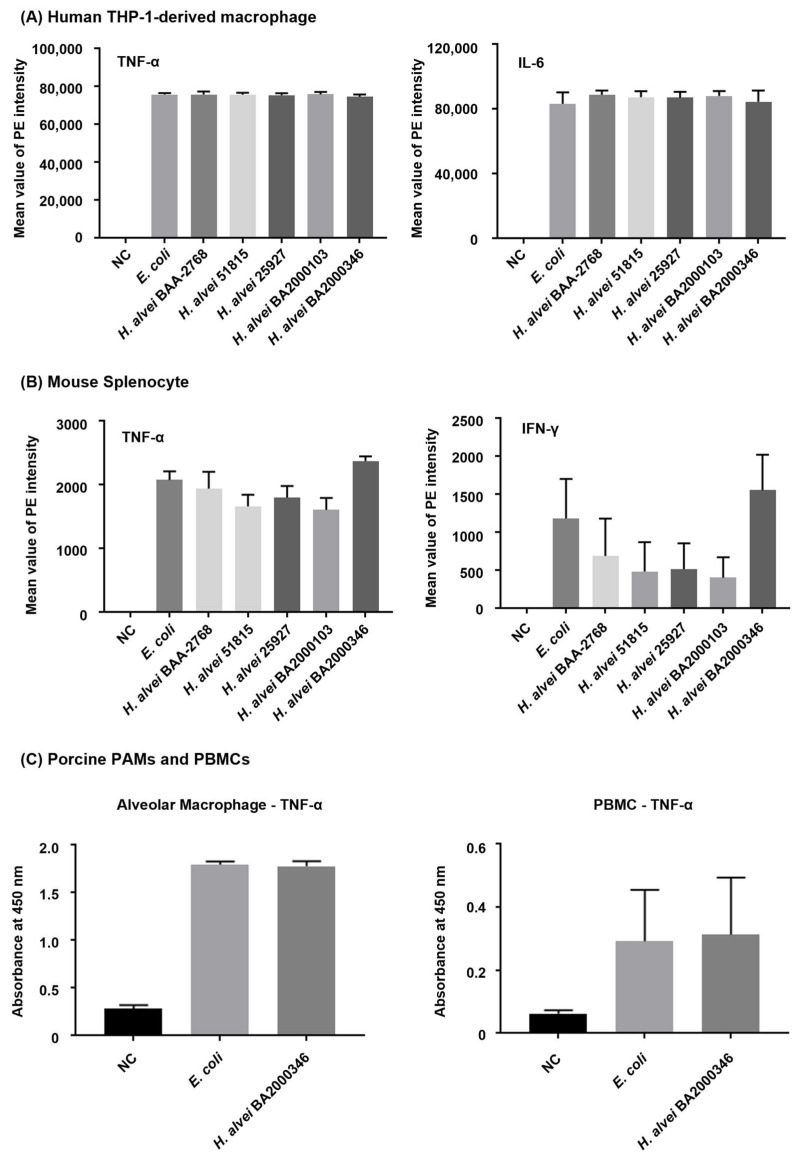
Comparative evaluation of immune responses induced by various *H. alvei*-derived LPS in human and animal immune cells. (**A**) Human THP-1-derived macrophages (1 × 10^6^ cells/mL), (**B**) mouse splenocytes (1 × 10^6^ cells/mL) and (**C**) porcine PBMCs (4 × 10^5^ cells/mL) and PAMs (1 × 10^6^ cells/mL) were stimulated with various *H. alvei* LPS (100 ng/mL) for 24 h. Cytokine levels in the culture supernatants were measured using a CBA assay or ELISA. Data are presented as mean ± SD ((**A**,**B**): *n* = 3; (**C**): *n* = 2).

In human THP-1-derived macrophages, all five tested *H. alvei* LPS preparations induced IL-6 and TNF-α expression at levels comparable to the *E. coli* O111:B4 positive control (Figure 3A). In murine splenocytes, LPS stimulation broadly increased TNF-α and IFN-γ secretion. While four *H. alvei* strains elicited slightly lower cytokine levels than *E. coli* O111:B4, the BA2000346 strain distinctly outperformed the control, inducing 1.14-fold higher TNF-α and 1.32-fold higher IFN-γ expression (Figure 3B). Consistently, in porcine peripheral blood mononuclear cells (PBMCs) and pulmonary alveolar macrophages (PAMs), BA2000346 LPS induced TNF-α levels equivalent to those of *E. coli* O111:B4 (Figure 3C). Collectively, these data highlight that BA2000346 LPS provides a preliminary indication of immunostimulatory activity across multiple mammalian species under conditions tested.

### 3.4. A Prototype Squalene-Based O/W Emulsion Containing H. alvei LPS Enhances Antigen-Specific Humoral and Cellular Immunity In Vivo

**Figure 4:**
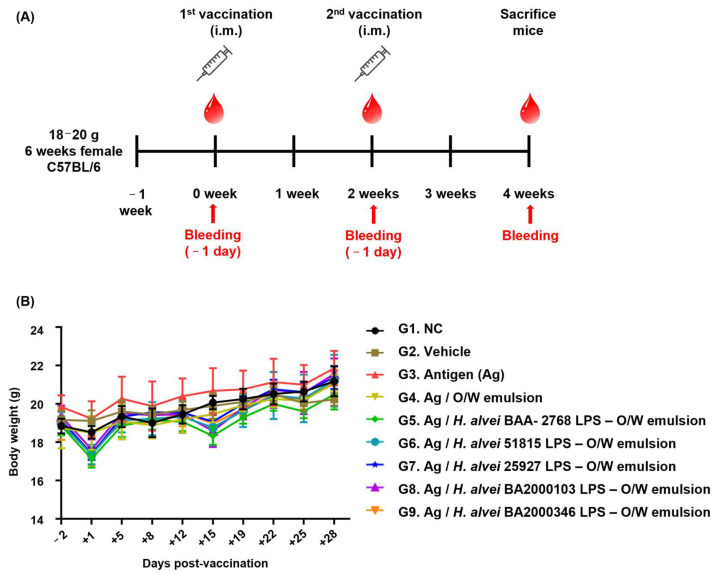
Preliminary tolerability assessment of O/W emulsion-based adjuvanted vaccines containing *H. alvei* LPS. (**A**) C57BL/6N mice were immunized twice with OVA antigen formulated with O/W emulsion containing various *H. alvei* LPS. (**B**) Body weight was monitored throughout the vaccination period. Data are shown as mean ± SD (*n* = 4 per group).

To evaluate the in vivo translational potential of *H. alvei* LPS as a vaccine adjuvant, LPS from various strains was formulated into a prototype squalene-based O/W emulsion and co-administered with ovalbumin (OVA) in a murine model (Figure 4A). Preliminary tolerability was initially assessed based on post-immunization body weight monitoring. Mice receiving *H. alvei* LPS-formulated adjuvants (Groups 5–9) exhibited only transient weight loss that fully recovered over time. Importantly, following the second (boost) injection, no severe systemic adverse responses or local injection-site inflammation were observed during the monitoring period (Figure 4B).

**Figure 5:**
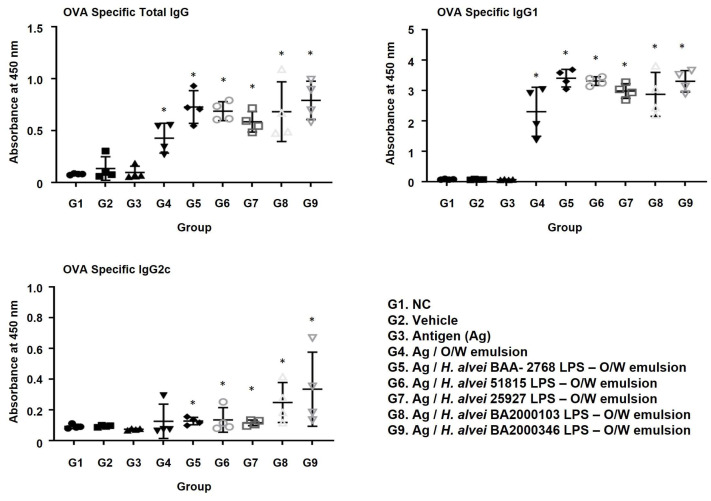
Analysis of antigen-specific antibody responses following vaccination with O/W emulsion-adjuvanted formulations containing *H. alvei* LPS. Sera collected at two weeks after the second immunization (DPV 28) were diluted 1:40,000 and analyzed for OVA-specific total IgG, IgG1, and IgG2c levels. Data are presented as mean ± SD (*n* = 4 biological replicates per group). Statistical significance was determined using the Kruskal–Wallis test followed by the Mann–Whitney test (* *p* < 0.05). Comparisons were made against the antigen-only group (G3). Due to the exploratory nature of the study, post hoc statistical comparisons were conducted without corrections for multiple comparisons.

Immunologically, the standalone O/W emulsion (Group 4) demonstrated an intrinsic ability to enhance antibody responses compared to the antigen-only group (Group 3) (Figure 5). Notably, incorporating *H. alvei* LPS into the O/W emulsion (Groups 5–9) significantly amplified antibody titers, with the most pronounced responses observed in the BA2000346-formulated group (Group 9). In this group, total IgG titers were elevated 8.21-fold relative to Group 3. Furthermore, IgG1 (Th2-associated) and IgG2c (Th1-associated) titers significantly increased by 48.07-fold and 4.55-fold, respectively (*p* < 0.05), surpassing the humoral responses induced by other strains.

**Figure 6:**
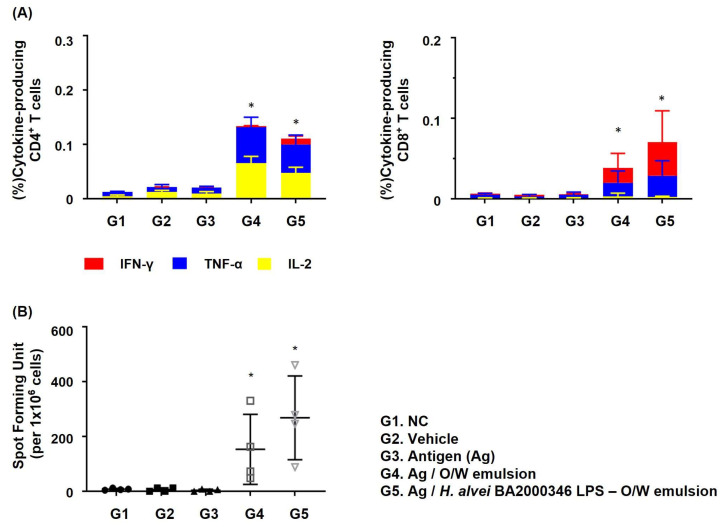
O/W emulsion containing *H. alvei* LPS enhances cellular immune responses beyond the effects of the emulsion alone. Splenocytes were collected at two weeks after the second immunization (DPV 28). (**A**) The percentages of antigen-specific cytokine (IFN-γ, TNF-α, and IL-2)-producing CD4 ^+^ and CD8^+^ T cells were determined by ICS assay following restimulation with OVA (50 μg/mL) for 15 h. (**B**) IFN-γ-secreting cells were quantified by ELISpot assay after restimulation with OVA_257–264_ (SIINFEKL, 10 μg/mL) for 20 h. Data are presented as mean ± SD (*n* = 4 biological replicates per group). Statistical significance was determined using the Kruskal–Wallis test followed by the Mann–Whitney test (* *p* < 0.05). Comparisons were made against the antigen-only group (G3). Due to the exploratory nature of the study, post hoc statistical comparisons were conducted without corrections for multiple comparisons.

Beyond humoral immunity, the *H. alvei* LPS-adjuvanted O/W formulation significantly enhanced cellular immune responses. Intracellular cytokine staining (ICS) revealed that the frequencies of antigen-specific CD4^+^ and CD8^+^ T cells expressing IFN-γ, TNF-α, and IL-2 were significantly elevated in the standalone O/W emulsion group (Group 4) and the BA2000346 LPS-formulated group (designated as Group 5 in this specific cellular assay) compared to the antigen-only control (*p* < 0.05). Specifically, relative to the antigen-only control (Group 3), the BA2000346 LPS-formulated group exhibited a 6.68-fold increase in TNF-α^+^ CD8^+^ T cells and a 51.15-fold increase in IFN-γ^+^ CD8^+^ T cells (Figure 6A). Furthermore, while the addition of BA2000346 LPS to the O/W emulsion (Group 5) showed a trend towards higher frequencies of IFN-γ^+^ CD8^+^ T cells compared to the O/W emulsion alone (Group 4), this difference did not reach statistical significance (*p* > 0.05). Consistently, IFN-γ ELISpot analysis demonstrated 43.71-fold and 76.57-fold increases in the frequency of antigen-specific IFN-γ-secreting cells in Groups 4 and 5, respectively, compared to the control (Figure 6B, *p* < 0.05), although the difference between Group 4 and Group 5 was similarly not statistically significant. Taken together, these results suggest that the *H. alvei* LPS/emulsion system acts as an exploratory adjuvant platform to stimulate cellular and humoral immune responses, requiring confirmation in adequately powered studies.

## 4. Discussion

Vaccinology has traditionally emphasized the selection of optimal antigen types—whether live-attenuated, inactivated, recombinant protein, peptide, DNA, or mRNA—alongside the identification of appropriate vaccine strains. Although the critical role of adjuvants is increasingly recognized, they are still frequently relegated to being secondary formulation components rather than key determinants of protective efficacy. However, for pathogens characterized by high genetic variability and immune evasion, such as SARS-CoV-2 in humans [16] or porcine reproductive and respiratory syndrome virus (PRRSV) in swine [17,18], antibody-mediated immunity alone is fundamentally insufficient. In these scenarios, including emerging zoonotic infections, strategies capable of simultaneously eliciting strong humoral and robust cellular immune responses are critically required. Furthermore, as potential, long-term speculative applications, the use of potent adjuvants could be indispensable for activating antigen-specific cytotoxic T cells and overcoming the immunosuppressive tumor microenvironments in therapeutic cancer interventions [19].

To meet these pressing needs, considerable efforts have been devoted to discovering and characterizing adjuvants that preferentially drive cellular immunity. These include TLR agonists (e.g., Pam3CSK4, poly I:C, MPL, GLA), STING agonists (e.g., c-di-GMP, c-di-AMP), and saponin-based systems like QS-21 or Matrix-M [20,21]. Among these, TLR4 remains a particularly attractive target (e.g., Monophosphoryl Lipid A in GSK’s AS01, or Glucopyranosyl Lipid A by IDRI) [20] due to its profound capacity to induce pro-inflammatory cytokines and chemokines that shape Th1 and cytotoxic T cell responses. While TLR4 signaling and its downstream cascades (MyD88–NF-κB and TRIF–IRF3) are evolutionarily conserved across mammals—including humans, mice, pigs, and dogs [22]—genetic variations in the TLR4 extracellular domain can significantly influence ligand recognition [22]. Therefore, validating candidate TLR4 agonists across multiple species is an essential prerequisite for their application as broad-spectrum or veterinary adjuvants under a ‘One Health’ paradigm.

In this context, our results demonstrate that *H. alvei* LPS activates immune cells from humans, mice, and pigs. This indicates that its receptor engagement is sufficiently conserved to provide a preliminary indication of cross-species stimulatory potential. Rather than establishing broad cross-species adjuvant applicability, these preliminary observations support further investigation into the suitability of *H. alvei* LPS as an exploratory immunostimulatory component.

Historically, LPS from Gram-negative bacteria has been recognized for its immunostimulatory properties; notably, the first human vaccine to incorporate LPS utilized the pathogenic *Salmonella minnesota* R595 strain [23]. Subsequent research heavily focused on the chemical detoxification of lipid A (e.g., MPL, GLA) or the synthesis of lipid A analogs to mitigate endotoxicity [24,25]. In contrast to LPS derived from highly pathogenic bacteria, LPS from commensal or non-pathogenic species typically induces a more modulated inflammatory cascade, offering preliminary tolerability based on body-weight monitoring and visual clinical observation while maintaining adjuvant potential [11]. This paradigm shift has catalyzed growing interest in commensal-derived LPS as a novel class of natural adjuvants.

*H. alvei* is a well-known commensal bacterium residing in the gastrointestinal tract of humans and animals, and is occasionally isolated from fermented foods such as cheese and processed meats [26,27,28]. Certain strains have even been investigated as probiotics due to their beneficial metabolic properties [29,30]. Immunologically, the literature reports indicate that the LPSs of specific *H. alvei* strains possess an α-linked mannose within their O-antigen region (mannosylated LPS), which is recognized by the C-type lectin receptor Dectin-2 [15]. It must be noted that the exact structural composition of the BA2000346 LPS preparation itself was not structurally characterized in this study. In the absence of mass spectrometry or NMR data, any assumption that BA2000346 LPS is mannosylated remains strictly speculative. However, our reporter cell assays demonstrate that BA2000346 LPS exhibits both TLR4 and Dectin-2 agonist activity in vitro. While TLR4 activation drives pro-inflammatory cytokines [31] and Dectin-2 engagement triggers Syk–NF-κB signaling [32], these assays support receptor activation in engineered systems but do not demonstrate that the adjuvant effect in vivo is mechanistically dependent on both receptors. Rather, these reporter assay findings raise the possibility that simultaneous engagement of TLR4 and Dectin-2 may contribute to the observed immunostimulatory profile, but this hypothesis requires confirmation in receptor-specific models (e.g., target-specific receptor blockade or knock-out in vivo models).

By systematically comparing LPS from multiple *H. alvei* strains, we identified that, while all preparations activated immune cells, BA2000346 LPS elicited higher IFN-γ production, consistent with its in vitro receptor activity. The observed discrepancy in bioactivity among the isolates suggests structural heterogeneity within the *H. alvei* species. We hypothesize that these functional differences stem from variations in their respective O-antigen compositions or distinct lipid A acylation patterns. When formulated into a prototype squalene-based O/W emulsion, this LPS was associated with increases in both humoral and cellular adaptive immunity. As demonstrated by our exploratory in vivo and intracellular cytokine staining (ICS) analysis, the formulation induced higher titers of antigen-specific IgG1 and IgG2c, alongside an expansion of multi-cytokine-producing CD4^+^ and CD8^+^ T cells. These preliminary findings suggest that commensal-derived *H. alvei* LPS, when effectively delivered, has the potential to act as a hypothesis-generating platform to stimulate cellular and humoral immune responses, requiring further confirmation in adequately powered studies.

Despite the promising preliminary dataset of *H. alvei* BA2000346 LPS, this proof-of-concept study has several limitations that warrant future investigation. First, due to the limited sample yield from the small-scale extraction, orthogonal purity assessments (e.g., Coomassie Brilliant Blue staining or BCA assays) were not performed to definitively rule out trace protein contamination, leaving silver staining as the sole preliminary indicator of purity. However, given the exceptionally low working concentrations utilized in our functional assays (e.g., 10–100 ng/mL in vitro and 1 μg/dose in vivo), the absolute quantity of any potential trace contaminants introduced would be negligible. Thus, it is unlikely that such trace impurities were the primary drivers of the observed immunological responses. Second, while functional assays indicate TLR4 and Dectin-2 agonist activity in vitro, we lack direct structural elucidation (e.g., nuclear magnetic resonance or mass spectrometry) to definitively confirm and quantify the exact proportion of α-linked mannose in the O-antigen of this specific strain. Future structural–functional mapping, alongside target-specific receptor blockade or knock-out in vivo models, is mandatory to map the exact mechanistic dependencies. Third, the exact degree of immunological enhancement cannot be definitively inferred or compared without extensive dose-titration of the LPS, which remains a weakness of the current experimental design. Fourth, the safety evaluation is highly preliminary, limited to preliminary tolerability based on body-weight monitoring and visual clinical observation. Comprehensive toxicological evaluations, including hematology data, serum inflammatory markers, organ weights, histopathology, local reactogenicity scoring, and dose-escalation safety assessments, remain vital requirements for future evaluation phases. Fifth, we explicitly state that the current emulsion characterization is preliminary and incomplete. While basic physicochemical properties (pH and particle size) of the prototype emulsion have been established, comprehensive characterizations including the exact method of emulsification, zeta potential, polydispersity index (PDI), short- or long-term stability, and batch-to-batch reproducibility remain a priority for upcoming formulation optimization studies. Sixth, the small sample sizes (e.g., *n* = 4 per group for in vivo experiments) limit the reliability of the statistical comparisons; therefore, the humoral and cellular immune response data should be strictly interpreted as exploratory observations requiring confirmation in adequately powered studies. Finally, as this proof-of-concept study utilized a model antigen (OVA) in mice, definitive translation requires rigorous pathogen challenge models, durability assessments, and extensive dose-optimization trials to validate its efficacy with clinically relevant antigens. Furthermore, while the current study evaluated the baseline immunological enhancement over the standalone O/W emulsion, future investigations must include head-to-head comparisons with established commercial adjuvants (e.g., Alum, AddaVax, or MPLA) to definitively benchmark the relative potency of this platform. Additionally, in this initial screening, humoral responses were evaluated at a standardized specific serum dilution rather than through full endpoint titrations or absolute gravimetric standardization, which limits precise quantitative comparisons with responses induced in other studies. Overall, rather than drawing definitive translational conclusions, the present findings may justify further investigation in disease-relevant antigen and challenge models.

## 5. Conclusions

This proof-of-concept study demonstrates that *H. alvei* strain BA2000346-derived LPS possesses the intrinsic capacity to exhibit TLR4 and Dectin-2 agonist activity in reporter system in vitro, providing a preliminary indication of cross-species stimulatory potential. Given its commensal origin, preliminary tolerability based on body-weight monitoring and visual clinical observation, and its ability to orchestrate both humoral and cellular immunity when synergized with a prototype O/W emulsion, *H. alvei* LPS represents an exploratory adjuvant platform for model antigens. Rather than drawing definitive conclusions, our findings may justify further investigation in disease-relevant antigen and challenge models to fully elucidate its translational potential across human and veterinary medicine.

## Data Availability

The datasets supporting the findings of this study are available from the corresponding author upon reasonable request.

## Supplementary Materials

The following supporting information can be downloaded at: https://www.mdpi.com/article/10.3390/vaccines14070557/s1, Figure S1: Purity assessment of extracted *Hafnia alvei* lipopolysaccharides (LPSs). LPS preparations extracted from various *H. alvei* strains were resolved by SDS-PAGE and visualized using a Pierce Silver Stain Kit. The gel demonstrates the successful extraction of LPS and the absence of detectable protein contamination, confirming the high purity of the LPS samples utilized for the in vitro and in vivo immunological assays; Figure S2: Representative flow cytometry gating strategy for evaluating cellular immune responses. Splenocytes harvested from immunized mice were restimulated with OVA (50 μg/mL) for 15 h and analyzed by intracellular cytokine staining (ICS). The sequential gating strategy was performed as follows: (1) identification of the general lymphocyte population based on forward and side scatter (FSC-A vs. SSC-A); (2) doublet exclusion to gate single cells (FSC-A vs. FSC-H); and (3) exclusion of dead cells utilizing a viability dye. From the viable single-cell population, CD4^+^ and CD8^+^ T cell subsets were identified. Subsequent gating was applied to quantify the frequencies of antigen-specific T cells producing intracellular cytokines (IFN-γ, TNF-α, and IL-2).

## Abbreviations

The following abbreviations are used in this manuscript:

LPS	Lipopolysaccharide
TLR4	Toll-like receptor 4
O/W	Oil-in-water
Alum	Aluminum salts
Th2	Type 2 helper T
Th1	Type 1 helper T
PRRs	Pattern recognition receptors
TLRs	Toll-like receptors
CLRs	C-type lectin receptors
