# Supplementary material for: Immunogenicity of an Oil-in-Water Emulsion Containing Hafnia Alvei-Derived Lipopolysaccharide, with TLR4 and Dectin-2 Agonist Activity In Vitro
Source: Vaccines (Basel). 2026 Jun 25;14(7):557. doi: 10.3390/vaccines14070557 (PMC13417346; doi:10.3390/vaccines14070557)
Supplement: Supplementary file 1 [file vaccines-14-00557-s001.zip › Supplementary Figure S1.pdf]

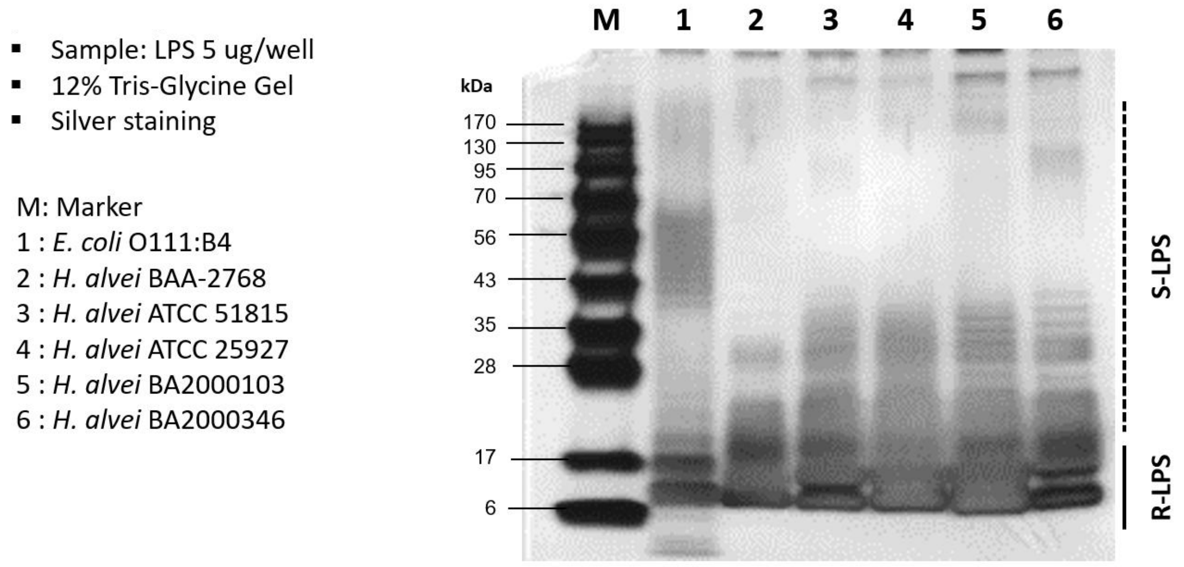

Supplementary Figure S1. Purity assessment of extracted *Hafnia alvei* lipopolysaccharides (LPS). LPS preparations extracted from various *H. alvei* strains were resolved by SDS-PAGE and visualized using a Pierce Silver Stain Kit. The gel demonstrates the successful extraction of LPS and the absence of detectable protein contamination, confirming the high purity of the LPS samples utilized for the *in vitro* and *in vivo* immunological assays.
