# Supplementary material for: Immunogenicity of an Oil-in-Water Emulsion Containing Hafnia Alvei-Derived Lipopolysaccharide, with TLR4 and Dectin-2 Agonist Activity In Vitro
Source: Vaccines (Basel). 2026 Jun 25;14(7):557. doi: 10.3390/vaccines14070557 (PMC13417346; doi:10.3390/vaccines14070557)
Supplement: Supplementary file 1 [file vaccines-14-00557-s001.zip › Supplementary Figure S2.pdf]

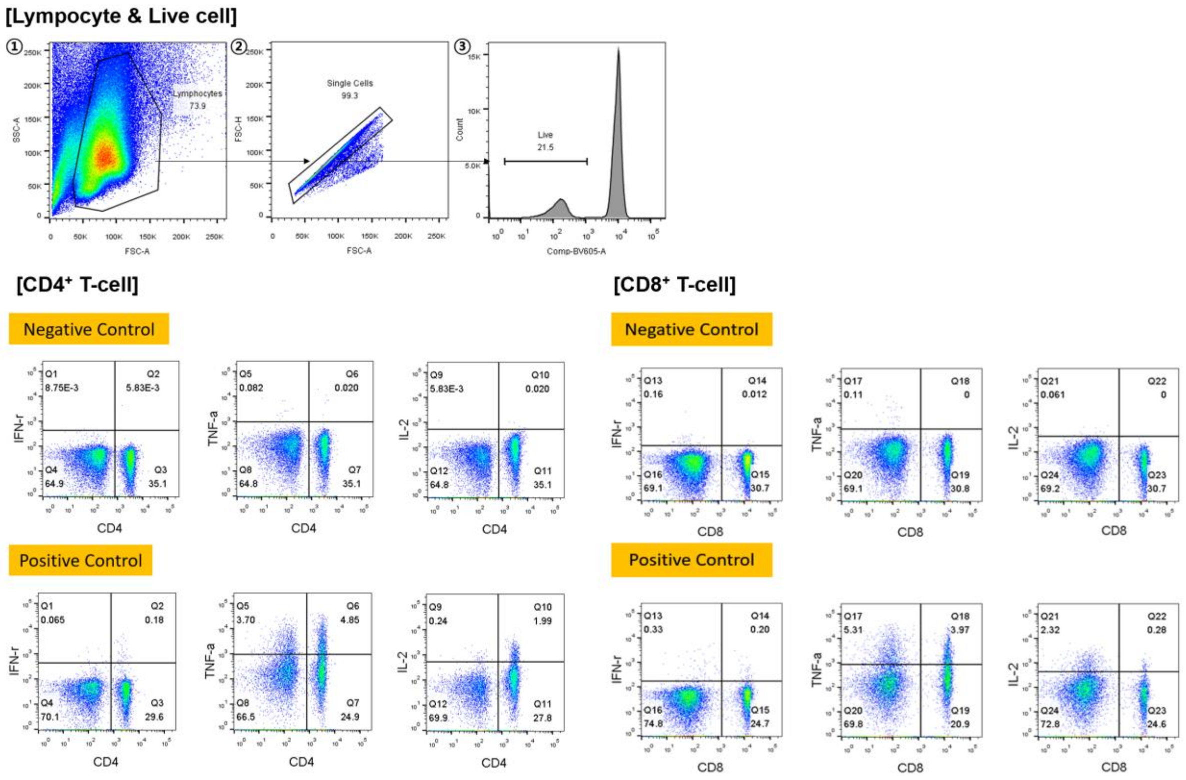

Supplementary Figure S2. Representative flow cytometry gating strategy for evaluating cellular immune responses. Splenocytes harvested from immunized mice were restimulated with OVA (50 μg/mL) for 15 h and analyzed by intracellular cytokine staining (ICS). The sequential gating strategy was performed as follows: (1) identification of the general lymphocyte population based on forward and side scatter (FSC-A vs. SSC-A); (2) doublet exclusion to gate single cells (FSC-A vs. FSC-H); and (3) exclusion of dead cells utilizing a viability dye. From the viable single-cell population, CD4+ and CD8+ T cell subsets were identified. Subsequent gating was applied to quantify the frequencies of antigen-specific T cells producing intracellular cytokines (IFN-γ, TNF-α, and IL-2).
